# Supplementary material for: Perinatal outcomes of symptomatic chikungunya, dengue and Zika infection during pregnancy in Brazil: a registry-based cohort study
Source: Nat Commun. 2025 Aug 5;16:7207. doi: 10.1038/s41467-025-62640-x (PMC12325709; doi:10.1038/s41467-025-62640-x)
Supplement: Supplementary file 1 — Supplementary Information [file 41467_2025_62640_MOESM1_ESM.pdf]

Supplement Information to: Cerqueira-Silva T, Perinatal outcomes of symptomatic chikungunya, dengue and Zika infection during pregnancy in Brazil: a registry-based cohort study. Nature Communications 2025

Table of Contents

Supplementary Table 1: Baseline characteristics of singleton live births.. 2

Supplementary Table 2: P-values from Heterogeneity test (Cochran's Q) of the effect by trimester for each virus. 4

Supplementary Table 3: Estimated crude and adjusted hazard ratio by exposure and outcome in the women with confirmed symptomatic infection by clinical or laboratory diagnosis. The Schoenfeld residuals test was based on the analysis using missing indicator. 5

Supplementary Table 4: Estimated crude and adjusted hazard ratio by exposure and outcome in the women with confirmed symptomatic infection only by laboratory diagnosis. The Schoenfeld residuals test was based on the analysis using missing indicator. 6

Supplementary Table 5: Estimated crude and adjusted hazard ratio by exposure, trimester and outcome in the women with confirmed symptomatic infection by clinical or laboratory diagnosis. The Schoenfeld residuals test was based on the analysis using missing indicator. 7

Supplementary Table 6: Estimated crude and adjusted hazard ratio by exposure, trimester and outcome in the women with confirmed symptomatic infection only by laboratory diagnosis. The Schoenfeld residuals test was based on the analysis using missing indicator. 10

Supplementary Table 7: Estimated risk ratio of neonatal death (<28 days) by exposure in the women with confirmed symptomatic infection by clinical or laboratory diagnosis. Analysis using missing indicator. 13

Supplementary Table 8: Estimated risk ratio of neonatal death (<28 days) by exposure in the women with confirmed symptomatic infection only by laboratory diagnosis. Analysis using missing indicator. 13

Supplementary Table 9: Estimated adjusted hazard ratios (birth outcomes) and adjusted risk ratios (neonatal death) using complete case analysis. 14

Supplementary Table 10: Distribution of congenital anomalies by exposure. 16

Supplementary Table 11: Distribution of deaths (ICD-10) by exposure. 18

Supplementary Figure 1: Exposure timelines for preterm analysis. Each line represents one pregnancy, and the line's colour represents the infection status for one arbovirus (time-dependent exposure). A red X indicates occurrence of an adverse outcome 19

Supplementary Figure 2: Estimated adjusted hazard ratio (birth outcomes) and adjusted risk ratio (neonatal death), comparing groups exposed and unexposed to arbovirus infection during pregnancy by outcome. "Bias" estimates are derived from the model, classifying the live born as exposed since conception if their mother had an infection at any point during the pregnancy, i.e. not using time-varying exposure. Errors bars represent the 95% confidence interval. The numbers of individuals in each group are present in the Supplementary Table 3. 20

Supplementary Figure 3 – Performance Linkage. Linkage between the Live Birth information System and arbovirus notification information system 21

STROBE Statement—checklist of items that should be included in reports of observational studies 22

Supplementary Table 1: Baseline characteristics of singleton live births

Characteristic		Unexposed, N = 6,959,911	Chikungunya, N = 6,066	Dengue, N = 19,022	Zika, N = 8,396	Overall, N = 6,993,395
**Age - mother, median (IQR)**		27 (22, 32)	26 (21, 31)	26 (21, 31)	27 (22, 32)	27 (22, 32)
**Age - mother**						
	<20	1,018,301 (14.6%)	1,058 (17.5%)	3,264 (17.2%)	1,240 (14.8%)	1,023,863 (14.6%)
	20-34	4,833,782 (69.5%)	4,218 (69.5%)	13,358 (70.2%)	5,946 (70.8%)	4,857,304 (69.5%)
	≥35	1,107,828 (15.9%)	790 (13.0%)	2,400 (12.6%)	1,210 (14.4%)	1,112,228 (15.9%)
**Year of pregnancy**						
	2015	1,668,960 (24.0%)	1,682 (27.7%)	8,914 (46.9%)	6,609 (78.7%)	1,686,165 (24.1%)
	2016	1,651,835 (23.7%)	1,900 (31.3%)	2,666 (14.0%)	1,234 (14.7%)	1,657,635 (23.7%)
	2017	1,715,454 (24.6%)	1,408 (23.2%)	1,324 (7.0%)	312 (3.7%)	1,718,498 (24.6%)
	2018	1,670,433 (24.0%)	906 (14.9%)	4,677 (24.6%)	211 (2.5%)	1,676,227 (24.0%)
	2019	253,229 (3.6%)	170 (2.8%)	1,441 (7.6%)	30 (0.4%)	254,870 (3.6%)
**Geographic region of residence**						
	North	636,595 (9.1%)	411 (6.8%)	680 (3.6%)	717 (8.5%)	638,403 (9.1%)
	Northeast	1,715,931 (24.7%)	3,970 (65.4%)	3,705 (19.5%)	796 (9.5%)	1,724,402 (24.7%)
	Southeast	3,501,294 (50.3%)	1,439 (23.7%)	10,201 (53.6%)	5,472 (65.2%)	3,518,406 (50.3%)
	South	322,083 (4.6%)	0 (0.0%)	575 (3.0%)	15 (0.2%)	322,673 (4.6%)
	Central west	784,008 (11.3%)	246 (4.1%)	3,861 (20.3%)	1,396 (16.6%)	789,511 (11.3%)
**Race/ethnicity**						
	White	2,249,165 (32.3%)	815 (13.4%)	5,871 (30.9%)	2,623 (31.2%)	2,258,474 (32.3%)
	Black	449,834 (6.5%)	230 (3.8%)	1,023 (5.4%)	384 (4.6%)	451,471 (6.5%)
	Indigenous	26,811 (0.4%)	12 (0.2%)	60 (0.3%)	6 (0.1%)	26,889 (0.4%)
	Mixed	3,920,721 (56.3%)	4,467 (73.6%)	11,033 (58.0%)	5,005 (59.6%)	3,941,226 (56.4%)
	Asian	36,319 (0.5%)	9 (0.1%)	103 (0.5%)	28 (0.3%)	36,459 (0.5%)
	Missing	277,061 (4.0%)	533 (8.8%)	932 (4.9%)	350 (4.2%)	278,876 (4.0%)
**Married/Stable union**		3,666,147 (52.7%)	3,012 (49.7%)	9,389 (49.4%)	3,469 (41.3%)	3,682,017 (52.6%)
	Missing	54,379 (0.8%)	65 (1.1%)	152 (0.8%)	68 (0.8%)	54,664 (0.8%)
**Years of schooling**						
	None to 3	116,234 (1.7%)	150 (2.5%)	277 (1.5%)	125 (1.5%)	116,786 (1.7%)
	4 to 7	934,198 (13.4%)	1,070 (17.6%)	2,565 (13.5%)	1,120 (13.3%)	938,953 (13.4%)
	8 to 11	4,207,533 (60.5%)	3,955 (65.2%)	12,884 (67.7%)	5,267 (62.7%)	4,229,639 (60.5%)
	≥12	1,633,737 (23.5%)	740 (12.2%)	3,124 (16.4%)	1,795 (21.4%)	1,639,396 (23.4%)
	Missing	68,209 (1.0%)	151 (2.5%)	172 (0.9%)	89 (1.1%)	68,621 (1.0%)
**Number of antenatal appointments**						
	None to 3	499,284 (7.2%)	322 (5.3%)	864 (4.5%)	345 (4.1%)	500,815 (7.2%)
	4 to 6	1,498,914 (21.5%)	1,321 (21.8%)	3,615 (19.0%)	1,564 (18.6%)	1,505,414 (21.5%)
	≥7	4,925,444 (70.8%)	4,399 (72.5%)	14,475 (76.1%)	6,422 (76.5%)	4,950,740 (70.8%)
	Missing	36,269 (0.5%)	24 (0.4%)	68 (0.4%)	65 (0.8%)	36,426 (0.5%)
**Delayed antenatal care (>3 months)**		1,182,189 (17.8%)	886 (15.6%)	2,623 (14.4%)	1,150 (14.4%)	1,186,848 (17.8%)
	Missing	330,165	376	839	383	331,763
**Previous pregnancies**						

None	4,191,614 (60.2%)	3,574 (58.9%)	10,560 (55.5%)	4,764 (56.7%)	4,210,512 (60.2%)
≥1	2,768,297 (39.8%)	2,492 (41.1%)	8,462 (44.5%)	3,632 (43.3%)	2,782,883 (39.8%)
**Previous fetal loss**	1,285,999 (18.5%)	1,275 (21.0%)	3,363 (17.7%)	1,651 (19.7%)	1,292,288 (18.5%)
Missing	383,632 (5.5%)	553 (9.1%)	958 (5.0%)	420 (5.0%)	385,563 (5.5%)
**Preterm birth (<37 weeks)**	692,170 (9.9%)	578 (9.5%)	1,722 (9.1%)	821 (9.8%)	695,291 (9.9%)
**Low birth weight (<2,500g)**	522,125 (7.5%)	404 (6.7%)	1,399 (7.4%)	654 (7.8%)	524,582 (7.5%)
**Apgar score 5', median (IQR)**	9.00 (9.00, 10.00)	9.00 (9.00, 10.00)	9.00 (9.00, 10.00)	9.00 (9.00, 10.00)	9.00 (9.00, 10.00)
Missing	79,419	88	218	51	79,776
**Low Apgar score 5' (<7)**	61,905 (0.9%)	69 (1.2%)	183 (1.0%)	84 (1.0%)	62,241 (0.9%)
Missing	79,419	88	218	51	79,776
**Congenital anomaly**	66,004 (0.9%)	55 (0.9%)	192 (1.0%)	145 (1.7%)	66,396 (0.9%)
**Weight for gestational age**					
AGA	5,387,318 (77.4%)	4,559 (75.2%)	14,991 (78.8%)	6,333 (75.4%)	5,413,201 (77.4%)
SGA	489,981 (7.0%)	456 (7.5%)	1,446 (7.6%)	673 (8.0%)	492,556 (7.0%)
LGA	1,082,612 (15.6%)	1,051 (17.3%)	2,585 (13.6%)	1,390 (16.6%)	1,087,638 (15.6%)
**Neonatal Death**	43,469 (0.6%)	53 (0.9%)	118 (0.6%)	51 (0.6%)	43,979 (0.6%)
**Pregnancy Arbovirus Symptomatic Infection**					
**Gestational age on symptom onset**	-	158 (97, 213)	134 (70, 199)	163 (106, 213)	147 (84, 205)
**Any arbovirus infection before pregnancy**	52,579 (0.8%)	66 (1.1%)	226 (1.2%)	58 (0.7%)	52,929 (0.8%)
**Diagnosis criteria**					
Laboratory		3,144 (51.8%)	6,316 (33.2%)	3,659 (43.6%)	13,119 (39.2%)
Clinical epidemiology		2,922 (48.2%)	12,706 (66.8%)	4,737 (56.4%)	20,365 (60.8%)
**Pre-conception Arbovirus Symptomatic Infection**					
**Previous chikungunya**	4,125 (0.1%)	10 (0.2%)	6 (<0.1%)	<5 (<0.1%)	4,143 (0.1%)
**Days from chikungunya to conception date**	419 (294, 559)	326 (280, 450)	406 (238, 631)	598 (567, 628)	419 (294, 559)
**Previous dengue**	45,901 (0.7%)	49 (0.8%)	215 (1.1%)	53 (0.6%)	46,218 (0.7%)
**Days from dengue to conception date**	468 (327, 604)	447 (240, 576)	403 (279, 593)	409 (240, 569)	468 (326, 604)
**Previous Zika**	3,082 (<0.1%)	9 (0.1%)	7 (<0.1%)	<5 (<0.1%)	3,101 (0.0%)
**Days from Zika to conception date**	445 (313, 571)	396 (251, 599)	191 (186, 368)	229 (223, 274)	444 (312, 571)

IQR = Interquartile Range, AGA = Adequate for gestational age, SGA = Small for gestational age, LGA = Large for gestational age. The raw percentages of the outcomes dependent of gestational age, such as preterm birth and low birth weight don't exhibit direct relationship with the estimated hazard ratio due to the infections being treated as a time-varying variable.

Supplementary Table 2: P-values from Heterogeneity test (Cochran's Q) of the effect by trimester for each virus.

	Chikungunya	*Dengue*	*Zika*
Preterm	0.14	0.02	0.84
Low birth weight	0.01	0.04	0.44
Small for gestational age	0.02	0.02	0.10
Large for gestational age	0.11	0.74	0.60
Congenital anomalies	<0.01	0.16	<0.001
Low Apgar 5'	0.04	0.04	0.513
Neonatal death	0.38	0.26	0.02

Supplementary Table 3: Estimated crude and adjusted hazard ratio by exposure and outcome in the women with confirmed symptomatic infection by clinical or laboratory diagnosis. The Schoenfeld residuals test was based on the analysis using missing indicator.

Outcome	*Group*	*Pregnancies weeks*	*No. pregnancies*	*Events*	*Rate 1,000 pregnancies/week*	*Crude Hazard Ratio*	*Adjusted Hazard Ratio (Missing indicator)*	*Schoenfeld residuals test p-value*
Preterm	Unexposed	255,285,029.43	6,993,395	692,170	2.71 (95% CI 2.70 to 2.72)			0.30
	Chikungunya	89,251.71	5,726	578	6.48 (95% CI 5.96 to 7.02)	1.11 (95% CI 1.02 to 1.21)	1.11 (95% CI 1.03 to 1.21)	
	Dengue	329,992.57	18,316	1,722	5.22 (95% CI 4.98 to 5.47)	1.01 (95% CI 0.96 to 1.06)	1.07 (95% CI 1.02 to 1.12)	
	Zika	117,939.14	7,938	821	6.96 (95% CI 6.50 to 7.45)	1.15 (95% CI 1.07 to 1.23)	1.25 (95% CI 1.17 to 1.34)	
Low birth weight	Unexposed	268,917,025.71	6,993,395	522,125	1.94 (95% CI 1.94 to 1.95)			0.60
	Chikungunya	101,698.57	6,066	404	3.97 (95% CI 3.60 to 4.37)	0.98 (95% CI 0.88 to 1.08)	1.11 (95% CI 1.01 to 1.22)	
	Dengue	367,609.86	19,022	1,399	3.81 (95% CI 3.61 to 4.01)	1.06 (95% CI 1.01 to 1.12)	1.10 (95% CI 1.04 to 1.16)	
	Zika	133,852.86	8,396	654	4.89 (95% CI 4.52 to 5.27)	1.18 (95% CI 1.09 to 1.27)	1.27 (95% CI 1.18 to 1.38)	
Small for gestational age	Unexposed	268,917,025.71	6,993,395	489,981	1.82 (95% CI 1.82 to 1.83)			0.49
	Chikungunya	101,698.57	6,066	456	4.48 (95% CI 4.08 to 4.91)	0.94 (95% CI 0.86 to 1.03)	1.04 (95% CI 0.95 to 1.14)	
	Dengue	367,609.86	19,022	1,446	3.93 (95% CI 3.73 to 4.14)	1.04 (95% CI 0.99 to 1.09)	1.00 (95% CI 0.95 to 1.06)	
	Zika	133,852.86	8,396	673	5.03 (95% CI 4.66 to 5.42)	1.15 (95% CI 1.07 to 1.24)	1.20 (95% CI 1.11 to 1.29)	
Large for gestational age	Unexposed	268,917,025.71	6,993,395	1,082,612	4.03 (95% CI 4.02 to 4.03)			<0.01
	Chikungunya	101,698.57	6,066	1,051	10.33 (95% CI 9.72 to 10.97)	1.09 (95% CI 1.02 to 1.15)	1.02 (95% CI 0.96 to 1.08)	
	Dengue	367,609.86	19,022	2,585	7.03 (95% CI 6.76 to 7.31)	0.87 (95% CI 0.84 to 0.91)	0.95 (95% CI 0.91 to 0.99)	
	Zika	133,852.86	8,396	1,390	10.38 (95% CI 9.85 to 10.94)	1.11 (95% CI 1.06 to 1.17)	1.12 (95% CI 1.06 to 1.18)	
Congenital anomalies	Unexposed	268,917,025.71	6,993,395	66,004	0.25 (95% CI 0.24 to 0.25)			0.45
	Chikungunya	101,698.57	6,066	55	0.54 (95% CI 0.41 to 0.70)	0.93 (95% CI 0.71 to 1.21)	1.06 (95% CI 0.81 to 1.38)	
	Dengue	367,609.86	19,022	192	0.52 (95% CI 0.45 to 0.60)	1.07 (95% CI 0.93 to 1.23)	1.19 (95% CI 1.03 to 1.37)	
	Zika	133,852.86	8,396	145	1.08 (95% CI 0.92 to 1.27)	1.92 (95% CI 1.63 to 2.25)	2.36 (95% CI 2.00 to 2.78)	
Low Apgar 5'	Unexposed	265,869,737.14	6,913,619	61,905	0.23 (95% CI 0.23 to 0.23)			0.82
	Chikungunya	100,251.00	5,978	69	0.69 (95% CI 0.54 to 0.86)	1.35 (95% CI 1.06 to 1.71)	1.44 (95% CI 1.13 to 1.83)	

	Dengue	363,672.86	18,804	183	0.50 (95% CI 0.43 to 0.58)	1.16 (95% CI 1.00 to 1.34)	1.25 (95% CI 1.08 to 1.45)
	Zika	133,012.00	8,345	84	0.63 (95% CI 0.51 to 0.78)	1.27 (95% CI 1.03 to 1.57)	1.31 (95% CI 1.05 to 1.62)

*The estimated hazard for a model with time-varying covariates does not exhibit the same direct relationship with cumulative incidence as the standard Cox model. This discrepancy arises because cumulative incidence depends on the complete history of the time-varying covariate for all patients. Consequently, the hazard ratio estimates from these models will not correspond to a crude rate ratio calculated using events per person-time. CI = Robust Confidence Interval

Supplementary Table 4: Estimated crude and adjusted hazard ratio by exposure and outcome in the women with confirmed symptomatic infection only by laboratory diagnosis. The Schoenfeld residuals test was based on the analysis using missing indicator.

Outcome	*Group*	*Pregnancies weeks*	*No. pregnancies*	*Events*	*Rate 1,000 pregnancies/week*	*Crude Hazard Ratio*	*Adjusted Hazard Ratio (Missing indicator)*	*Schoenfeld residuals test p-value*
Preterm	Unexposed	254,879,056.00	6,973,030	692,170	2.72 (95% CI 2.71 to 2.72)			0.84
	Chikungunya	44,896.86	2,976	282	6.28 (95% CI 5.58 to 7.04)	1.04 (95% CI 0.92 to 1.17)	1.08 (95% CI 0.96 to 1.21)	
	Dengue	101,663.43	6,056	596	5.86 (95% CI 5.40 to 6.35)	1.06 (95% CI 0.98 to 1.15)	1.18 (95% CI 1.08 to 1.28)	
	Zika	50,984.00	3,480	354	6.94 (95% CI 6.24 to 7.69)	1.12 (95% CI 1.01 to 1.24)	1.26 (95% CI 1.13 to 1.40)	
Low birth weight	Unexposed	268,509,857.86	6,973,030	522,125	1.94 (95% CI 1.94 to 1.95)			0.92
	Chikungunya	51,419.86	3,144	185	3.60 (95% CI 3.10 to 4.14)	0.85 (95% CI 0.74 to 0.99)	1.00 (95% CI 0.86 to 1.15)	
	Dengue	113,534.57	6,316	484	4.26 (95% CI 3.89 to 4.65)	1.13 (95% CI 1.03 to 1.24)	1.22 (95% CI 1.11 to 1.33)	
	Zika	57,933.71	3,659	268	4.63 (95% CI 4.09 to 5.20)	1.10 (95% CI 0.97 to 1.23)	1.20 (95% CI 1.07 to 1.36)	
Small for gestational age	Unexposed	268,509,857.86	6,973,030	489,981	1.82 (95% CI 1.82 to 1.83)			0.09
	Chikungunya	51,419.86	3,144	223	4.34 (95% CI 3.79 to 4.93)	0.87 (95% CI 0.76 to 0.99)	0.96 (95% CI 0.85 to 1.10)	
	Dengue	113,534.57	6,316	445	3.92 (95% CI 3.57 to 4.30)	1.02 (95% CI 0.93 to 1.12)	0.99 (95% CI 0.90 to 1.09)	
	Zika	57,933.71	3,659	275	4.75 (95% CI 4.21 to 5.33)	1.12 (95% CI 0.99 to 1.26)	1.16 (95% CI 1.03 to 1.31)	
Large for gestational age	Unexposed	268,509,857.86	6,973,030	1,082,612	4.03 (95% CI 4.02 to 4.04)			0.22
	Chikungunya	51,419.86	3,144	522	10.15 (95% CI 9.31 to 11.05)	1.03 (95% CI 0.94 to 1.12)	0.96 (95% CI 0.88 to 1.05)	
	Dengue	113,534.57	6,316	866	7.63 (95% CI 7.13 to 8.15)	0.93 (95% CI 0.87 to 0.99)	0.97 (95% CI 0.91 to 1.04)	
	Zika	57,933.71	3,659	577	9.96 (95% CI 9.17 to 10.79)	1.06 (95% CI 0.98 to 1.15)	1.08 (95% CI 0.99 to 1.17)	
	Unexposed	268,509,857.86	6,973,030	66,004	0.25 (95% CI 0.24 to 0.25)			0.55

Congenital anomalies	Chikungunya	51,419.86	3,144	31	0.60 (95% CI 0.41 to 0.84)	1.00 (95% CI 0.70 to 1.42)	1.17 (95% CI 0.82 to 1.67)	
	Dengue	113,534.57	6,316	58	0.51 (95% CI 0.39 to 0.65)	1.02 (95% CI 0.79 to 1.32)	1.10 (95% CI 0.85 to 1.43)	
	Zika	57,933.71	3,659	63	1.09 (95% CI 0.84 to 1.38)	1.92 (95% CI 1.50 to 2.45)	2.34 (95% CI 1.82 to 3.00)	
Low Apgar 5'	Unexposed	265,467,842.86	6,893,512	61,905	0.23 (95% CI 0.23 to 0.24)			0.59
	Chikungunya	51,123.71	3,123	29	0.57 (95% CI 0.39 to 0.80)	1.07 (95% CI 0.75 to 1.55)	1.17 (95% CI 0.81 to 1.69)	
	Dengue	112,521.43	6,253	64	0.57 (95% CI 0.44 to 0.72)	1.28 (95% CI 1.00 to 1.64)	1.45 (95% CI 1.13 to 1.85)	
	Zika	57,697.00	3,644	33	0.57 (95% CI 0.40 to 0.79)	1.15 (95% CI 0.82 to 1.62)	1.25 (95% CI 0.89 to 1.76)	

*The estimated hazard for a model with time-varying covariates does not exhibit the same direct relationship with cumulative incidence as the standard Cox model. This discrepancy arises because cumulative incidence depends on the complete history of the time-varying covariate for all patients. Consequently, the hazard ratio estimates from these models will not correspond to a crude rate ratio calculated using events per person-time. CI = Robust Confidence Interval

Supplementary Table 5: Estimated crude and adjusted hazard ratio by exposure, trimester and outcome in the women with confirmed symptomatic infection by clinical or laboratory diagnosis. The Schoenfeld residuals test was based on the analysis using missing indicator.

Outcome	*Group*	*Pregnancies weeks*	*No. pregnancies*	*Events*	*Rate 1,000 pregnancies/week*	*Crude Hazard Ratio*	*Adjusted Hazard Ratio (Missing indicator)*	*Schoenfeld residuals test p-value*
Preterm	Unexposed	255,285,029.43	6,993,395	692,170	2.71 (95% CI 2.70 to 2.72)			0.12
	Chikungunya first trimester	43,146.57	1,534	174	4.03 (95% CI 3.46 to 4.66)	1.15 (95% CI 0.99 to 1.33)	1.17 (95% CI 1.01 to 1.36)	
	Chikungunya second trimester	38,621.86	2,529	255	6.60 (95% CI 5.82 to 7.45)	1.03 (95% CI 0.91 to 1.16)	1.02 (95% CI 0.90 to 1.16)	
	Chikungunya third trimester	7,483.29	1,663	149	19.91 (95% CI 16.88 to 23.28)	1.24 (95% CI 1.06 to 1.46)	1.23 (95% CI 1.05 to 1.45)	
	Dengue first trimester	198,111.57	6,847	664	3.35 (95% CI 3.10 to 3.61)	0.97 (95% CI 0.90 to 1.05)	1.03 (95% CI 0.96 to 1.11)	
	Dengue second trimester	112,218.57	7,175	686	6.11 (95% CI 5.67 to 6.58)	0.97 (95% CI 0.90 to 1.04)	1.04 (95% CI 0.96 to 1.12)	
	Dengue third trimester	19,662.43	4,294	372	18.92 (95% CI 17.06 to 20.91)	1.19 (95% CI 1.07 to 1.31)	1.21 (95% CI 1.10 to 1.35)	
	Zika first trimester	49,762.00	1,810	195	3.92 (95% CI 3.39 to 4.49)	1.09 (95% CI 0.95 to 1.26)	1.21 (95% CI 1.05 to 1.40)	
	Zika second trimester	57,379.14	3,750	425	7.41 (95% CI 6.72 to 8.13)	1.16 (95% CI 1.06 to 1.28)	1.28 (95% CI 1.16 to 1.40)	

	Zika third trimester	10,798.00	2,378	201	18.61 (95% CI 16.16 to 21.31)	1.17 (95% CI 1.02 to 1.35)	1.23 (95% CI 1.07 to 1.42)	
Low birth weight	Unexposed	268,917,025.71	6,993,395	522,125	1.94 (95% CI 1.94 to 1.95)			0.31
	Chikungunya first trimester	46,351.86	1,534	86	1.86 (95% CI 1.49 to 2.28)	0.73 (95% CI 0.59 to 0.91)	0.85 (95% CI 0.69 to 1.05)	
	Chikungunya second trimester	43,880.71	2,529	200	4.56 (95% CI 3.95 to 5.22)	1.05 (95% CI 0.91 to 1.20)	1.18 (95% CI 1.02 to 1.35)	
	Chikungunya third trimester	11,466.00	2,003	118	10.29 (95% CI 8.54 to 12.26)	1.11 (95% CI 0.93 to 1.33)	1.27 (95% CI 1.06 to 1.52)	
	Dengue first trimester	211,904.86	6,847	507	2.39 (95% CI 2.19 to 2.61)	0.98 (95% CI 0.90 to 1.07)	1.01 (95% CI 0.93 to 1.11)	
	Dengue second trimester	126,464.57	7,175	576	4.55 (95% CI 4.19 to 4.94)	1.08 (95% CI 0.99 to 1.17)	1.13 (95% CI 1.04 to 1.23)	
	Dengue third trimester	29,240.43	5,000	316	10.81 (95% CI 9.66 to 12.04)	1.19 (95% CI 1.07 to 1.33)	1.19 (95% CI 1.07 to 1.33)	
	Zika first trimester	53,210.71	1,810	168	3.16 (95% CI 2.70 to 3.66)	1.25 (95% CI 1.08 to 1.46)	1.37 (95% CI 1.18 to 1.60)	
	Zika second trimester	64,558.14	3,750	316	4.89 (95% CI 4.37 to 5.45)	1.15 (95% CI 1.03 to 1.28)	1.26 (95% CI 1.12 to 1.40)	
	Zika third trimester	16,084.00	2,836	170	10.57 (95% CI 9.06 to 12.24)	1.16 (95% CI 1.00 to 1.35)	1.22 (95% CI 1.05 to 1.42)	
Small for gestational age	Unexposed	268,917,025.71	6,993,395	489,981	1.82 (95% CI 1.82 to 1.83)			0.73
	Chikungunya first trimester	46,351.86	1,534	87	1.88 (95% CI 1.51 to 2.30)	0.70 (95% CI 0.57 to 0.87)	0.80 (95% CI 0.65 to 0.98)	
	Chikungunya second trimester	43,880.71	2,529	205	4.67 (95% CI 4.06 to 5.34)	1.03 (95% CI 0.90 to 1.18)	1.13 (95% CI 0.98 to 1.30)	
	Chikungunya third trimester	11,466.00	2,003	164	14.30 (95% CI 12.22 to 16.61)	1.00 (95% CI 0.86 to 1.16)	1.10 (95% CI 0.95 to 1.29)	
	Dengue first trimester	211,904.86	6,847	477	2.25 (95% CI 2.05 to 2.46)	0.93 (95% CI 0.85 to 1.02)	0.91 (95% CI 0.83 to 1.00)	
	Dengue second trimester	126,464.57	7,175	574	4.54 (95% CI 4.18 to 4.92)	1.11 (95% CI 1.02 to 1.21)	1.08 (95% CI 0.99 to 1.17)	
	Dengue third trimester	29,240.43	5,000	395	13.51 (95% CI 12.22 to 14.88)	1.08 (95% CI 0.98 to 1.19)	1.01 (95% CI 0.92 to 1.12)	
	Zika first trimester	53,210.71	1,810	163	3.06 (95% CI 2.62 to 3.56)	1.30 (95% CI 1.12 to 1.52)	1.38 (95% CI 1.18 to 1.61)	
	Zika second trimester	64,558.14	3,750	283	4.38 (95% CI 3.89 to 4.91)	1.08 (95% CI 0.96 to 1.22)	1.13 (95% CI 1.01 to 1.27)	
	Zika third trimester	16,084.00	2,836	227	14.11 (95% CI 12.36 to 16.03)	1.15 (95% CI 1.01 to 1.31)	1.17 (95% CI 1.03 to 1.34)	
Large for gestational age	Unexposed	268,917,025.71	6,993,395	1,082,612	4.03 (95% CI 4.02 to 4.03)			0.02
	Chikungunya first trimester	46,351.86	1,534	308	6.64 (95% CI 5.93 to 7.41)	1.22 (95% CI 1.09 to 1.37)	1.12 (95% CI 1.00 to 1.25)	

	Chikungunya second trimester	43,880.71	2,529	443	10.10 (95% CI 9.18 to 11.07)	1.07 (95% CI 0.97 to 1.17)	1.01 (95% CI 0.92 to 1.10)	
	Chikungunya third trimester	11,466.00	2,003	300	26.16 (95% CI 23.31 to 29.24)	0.99 (95% CI 0.89 to 1.11)	0.95 (95% CI 0.84 to 1.06)	
	Dengue first trimester	211,904.86	6,847	980	4.62 (95% CI 4.34 to 4.92)	0.89 (95% CI 0.84 to 0.95)	0.97 (95% CI 0.91 to 1.03)	
	Dengue second trimester	126,464.57	7,175	977	7.73 (95% CI 7.25 to 8.22)	0.86 (95% CI 0.81 to 0.92)	0.95 (95% CI 0.89 to 1.01)	
	Dengue third trimester	29,240.43	5,000	628	21.48 (95% CI 19.84 to 23.20)	0.86 (95% CI 0.79 to 0.92)	0.93 (95% CI 0.86 to 1.01)	
	Zika first trimester	53,210.71	1,810	314	5.90 (95% CI 5.27 to 6.58)	1.14 (95% CI 1.02 to 1.28)	1.15 (95% CI 1.03 to 1.28)	
	Zika second trimester	64,558.14	3,750	620	9.60 (95% CI 8.87 to 10.38)	1.09 (95% CI 1.01 to 1.18)	1.08 (95% CI 1.00 to 1.17)	
	Zika third trimester	16,084.00	2,836	456	28.35 (95% CI 25.83 to 31.03)	1.13 (95% CI 1.03 to 1.24)	1.14 (95% CI 1.04 to 1.25)	
Congenital anomalies	Unexposed	268,917,025.71	6,993,395	66,004	0.25 (95% CI 0.24 to 0.25)			0.93
	Chikungunya first trimester	46,351.86	1,534	13	0.28 (95% CI 0.15 to 0.46)	0.84 (95% CI 0.49 to 1.44)	0.94 (95% CI 0.55 to 1.63)	
	Chikungunya second trimester	43,880.71	2,529	14	0.32 (95% CI 0.18 to 0.52)	0.55 (95% CI 0.33 to 0.93)	0.64 (95% CI 0.38 to 1.08)	
	Chikungunya third trimester	11,466.00	2,003	28	2.44 (95% CI 1.65 to 3.46)	1.54 (95% CI 1.06 to 2.23)	1.74 (95% CI 1.20 to 2.52)	
	Dengue first trimester	211,904.86	6,847	82	0.39 (95% CI 0.31 to 0.48)	1.23 (95% CI 0.99 to 1.53)	1.34 (95% CI 1.08 to 1.66)	
	Dengue second trimester	126,464.57	7,175	60	0.47 (95% CI 0.36 to 0.60)	0.87 (95% CI 0.68 to 1.12)	0.98 (95% CI 0.76 to 1.27)	
	Dengue third trimester	29,240.43	5,000	50	1.71 (95% CI 1.28 to 2.23)	1.14 (95% CI 0.87 to 1.51)	1.29 (95% CI 0.98 to 1.70)	
	Zika first trimester	53,210.71	1,810	80	1.50 (95% CI 1.20 to 1.86)	4.78 (95% CI 3.84 to 5.95)	6.19 (95% CI 4.96 to 7.72)	
	Zika second trimester	64,558.14	3,750	36	0.56 (95% CI 0.39 to 0.76)	1.03 (95% CI 0.75 to 1.43)	1.26 (95% CI 0.91 to 1.76)	
	Zika third trimester	16,084.00	2,836	29	1.80 (95% CI 1.22 to 2.54)	1.20 (95% CI 0.84 to 1.73)	1.44 (95% CI 1.00 to 2.07)	
Low Apgar 5'	Unexposed	265,869,737.14	6,913,619	61,905	0.23 (95% CI 0.23 to 0.23)			0.82
	Chikungunya first trimester	45,625.00	1,509	10	0.22 (95% CI 0.11 to 0.38)	0.69 (95% CI 0.37 to 1.29)	0.77 (95% CI 0.41 to 1.43)	
	Chikungunya second trimester	43,299.29	2,496	41	0.95 (95% CI 0.69 to 1.27)	1.79 (95% CI 1.32 to 2.44)	1.86 (95% CI 1.36 to 2.53)	
	Chikungunya third trimester	11,326.71	1,973	18	1.59 (95% CI 0.96 to 2.44)	1.30 (95% CI 0.82 to 2.06)	1.40 (95% CI 0.88 to 2.23)	
	Dengue first trimester	209,735.00	6,775	60	0.29 (95% CI 0.22 to 0.36)	0.96 (95% CI 0.74 to 1.24)	1.04 (95% CI 0.81 to 1.34)	
	Dengue second trimester	125,048.86	7,096	70	0.56 (95% CI 0.44 to 0.70)	1.12 (95% CI 0.89 to 1.42)	1.23 (95% CI 0.97 to 1.55)	

	Dengue third trimester	28,889.00	4,933	53	1.83 (95% CI 1.38 to 2.37)	1.61 (95% CI 1.23 to 2.11)	1.69 (95% CI 1.29 to 2.22)	
	Zika first trimester	52,862.00	1,798	20	0.38 (95% CI 0.24 to 0.57)	1.25 (95% CI 0.81 to 1.94)	1.28 (95% CI 0.82 to 1.98)	
	Zika second trimester	64,118.71	3,722	36	0.56 (95% CI 0.40 to 0.77)	1.13 (95% CI 0.81 to 1.57)	1.18 (95% CI 0.85 to 1.64)	
	Zika third trimester	16,031.29	2,825	28	1.75 (95% CI 1.18 to 2.48)	1.53 (95% CI 1.06 to 2.22)	1.55 (95% CI 1.07 to 2.25)	

*The estimated hazard for a model with time-varying covariates does not exhibit the same direct relationship with cumulative incidence as the standard Cox model. This discrepancy arises because cumulative incidence depends on the complete history of the time-varying covariate for all patients. Consequently, the hazard ratio estimates from these models will not correspond to a crude rate ratio calculated using events per person-time. CI = Robust Confidence Interval

Supplementary Table 6: Estimated crude and adjusted hazard ratio by exposure, trimester and outcome in the women with confirmed symptomatic infection only by laboratory diagnosis. The Schoenfeld residuals test was based on the analysis using missing indicator.

Outcome	*Group*	*Pregnancies weeks*	*No. pregnancies*	*Events*	*Rate 1,000 pregnancies/week*	*Crude Hazard Ratio*	*Adjusted Hazard Ratio (Missing indicator)*	*Schoenfeld residuals test p-value*
Preterm	Unexposed	254,879,056.00	6,973,030	692,170	2.72 (95% CI 2.71 to 2.72)			0.12
	Chikungunya first trimester	19,675.43	717	70	3.56 (95% CI 2.79 to 4.46)	0.98 (95% CI 0.77 to 1.24)	1.06 (95% CI 0.84 to 1.33)	
	Chikungunya second trimester	21,268.71	1,395	141	6.63 (95% CI 5.59 to 7.78)	1.03 (95% CI 0.87 to 1.21)	1.06 (95% CI 0.89 to 1.25)	
	Chikungunya third trimester	3,952.71	864	71	17.96 (95% CI 14.10 to 22.47)	1.13 (95% CI 0.89 to 1.42)	1.14 (95% CI 0.90 to 1.44)	
	Dengue first trimester	53,871.86	1,888	196	3.64 (95% CI 3.15 to 4.17)	1.04 (95% CI 0.91 to 1.20)	1.15 (95% CI 1.00 to 1.32)	
	Dengue second trimester	40,563.29	2,610	269	6.63 (95% CI 5.87 to 7.46)	1.05 (95% CI 0.93 to 1.18)	1.17 (95% CI 1.04 to 1.32)	
	Dengue third trimester	7,228.29	1,558	131	18.12 (95% CI 15.19 to 21.41)	1.14 (95% CI 0.96 to 1.35)	1.24 (95% CI 1.04 to 1.47)	
	Zika first trimester	18,668.86	694	74	3.96 (95% CI 3.13 to 4.94)	1.07 (95% CI 0.86 to 1.35)	1.23 (95% CI 0.98 to 1.55)	
	Zika second trimester	27,845.43	1,814	198	7.11 (95% CI 6.17 to 8.15)	1.12 (95% CI 0.97 to 1.28)	1.26 (95% CI 1.10 to 1.46)	
	Zika third trimester	4,469.71	972	82	18.35 (95% CI 14.66 to 22.61)	1.16 (95% CI 0.94 to 1.44)	1.26 (95% CI 1.01 to 1.57)	
	Unexposed	268,509,857.86	6,973,030	522,125	1.94 (95% CI 1.94 to 1.95)			0.59

Low birth weight	Chikungunya first trimester	21,203.86	717	33	1.56 (95% CI 1.08 to 2.15)	0.60 (95% CI 0.42 to 0.84)	0.74 (95% CI 0.53 to 1.04)	
	Chikungunya second trimester	24,173.86	1,395	97	4.01 (95% CI 3.27 to 4.86)	0.92 (95% CI 0.75 to 1.12)	1.06 (95% CI 0.87 to 1.29)	
	Chikungunya third trimester	6,042.14	1,032	55	9.10 (95% CI 6.90 to 11.72)	0.99 (95% CI 0.76 to 1.28)	1.12 (95% CI 0.86 to 1.46)	
	Dengue first trimester	57,454.57	1,888	151	2.63 (95% CI 2.23 to 3.07)	1.07 (95% CI 0.92 to 1.26)	1.17 (95% CI 0.99 to 1.37)	
	Dengue second trimester	45,477.71	2,610	223	4.90 (95% CI 4.29 to 5.58)	1.17 (95% CI 1.02 to 1.33)	1.26 (95% CI 1.11 to 1.44)	
	Dengue third trimester	10,602.29	1,818	110	10.38 (95% CI 8.56 to 12.44)	1.14 (95% CI 0.95 to 1.38)	1.21 (95% CI 1.00 to 1.46)	
	Zika first trimester	19,935.43	694	62	3.11 (95% CI 2.40 to 3.95)	1.22 (95% CI 0.95 to 1.56)	1.37 (95% CI 1.06 to 1.76)	
	Zika second trimester	31,339.29	1,814	140	4.47 (95% CI 3.77 to 5.25)	1.05 (95% CI 0.89 to 1.24)	1.16 (95% CI 0.98 to 1.38)	
	Zika third trimester	6,659.00	1,151	66	9.91 (95% CI 7.71 to 12.50)	1.10 (95% CI 0.86 to 1.39)	1.15 (95% CI 0.91 to 1.47)	
Small for gestational age	Unexposed	268,509,857.86	6,973,030	489,981	1.82 (95% CI 1.82 to 1.83)			0.12
	Chikungunya first trimester	21,203.86	717	42	1.98 (95% CI 1.44 to 2.64)	0.73 (95% CI 0.55 to 0.98)	0.84 (95% CI 0.63 to 1.13)	
	Chikungunya second trimester	24,173.86	1,395	94	3.89 (95% CI 3.15 to 4.73)	0.85 (95% CI 0.70 to 1.04)	0.94 (95% CI 0.77 to 1.15)	
	Chikungunya third trimester	6,042.14	1,032	87	14.40 (95% CI 11.58 to 17.64)	0.98 (95% CI 0.80 to 1.21)	1.07 (95% CI 0.87 to 1.31)	
	Dengue first trimester	57,454.57	1,888	123	2.14 (95% CI 1.78 to 2.54)	0.93 (95% CI 0.78 to 1.12)	0.92 (95% CI 0.77 to 1.11)	
	Dengue second trimester	45,477.71	2,610	187	4.11 (95% CI 3.55 to 4.73)	1.06 (95% CI 0.92 to 1.22)	1.03 (95% CI 0.90 to 1.19)	
	Dengue third trimester	10,602.29	1,818	135	12.73 (95% CI 10.70 to 15.00)	1.05 (95% CI 0.88 to 1.24)	1.01 (95% CI 0.85 to 1.20)	
	Zika first trimester	19,935.43	694	65	3.26 (95% CI 2.53 to 4.12)	1.52 (95% CI 1.19 to 1.94)	1.62 (95% CI 1.28 to 2.07)	
	Zika second trimester	31,339.29	1,814	123	3.92 (95% CI 3.27 to 4.66)	1.00 (95% CI 0.84 to 1.19)	1.04 (95% CI 0.87 to 1.24)	
	Zika third trimester	6,659.00	1,151	87	13.07 (95% CI 10.51 to 16.01)	1.08 (95% CI 0.88 to 1.34)	1.11 (95% CI 0.90 to 1.37)	
Large for gestational age	Unexposed	268,509,857.86	6,973,030	1,082,612	4.03 (95% CI 4.02 to 4.04)			0.39
	Chikungunya first trimester	21,203.86	717	143	6.74 (95% CI 5.70 to 7.91)	1.19 (95% CI 1.00 to 1.40)	1.08 (95% CI 0.92 to 1.27)	
	Chikungunya second trimester	24,173.86	1,395	244	10.09 (95% CI 8.88 to 11.41)	1.07 (95% CI 0.94 to 1.21)	1.00 (95% CI 0.88 to 1.13)	
	Chikungunya third trimester	6,042.14	1,032	135	22.34 (95% CI 18.78 to 26.33)	0.85 (95% CI 0.72 to 1.01)	0.81 (95% CI 0.68 to 0.96)	
	Dengue first trimester	57,454.57	1,888	277	4.82 (95% CI 4.28 to 5.41)	0.97 (95% CI 0.86 to 1.09)	1.00 (95% CI 0.89 to 1.12)	

	Dengue second trimester	45,477.71	2,610	369	8.11 (95% CI 7.31 to 8.97)	0.94 (95% CI 0.85 to 1.04)	1.00 (95% CI 0.90 to 1.10)	
	Dengue third trimester	10,602.29	1,818	220	20.75 (95% CI 18.13 to 23.61)	0.85 (95% CI 0.74 to 0.97)	0.90 (95% CI 0.79 to 1.03)	
	Zika first trimester	19,935.43	694	119	5.97 (95% CI 4.96 to 7.11)	1.18 (95% CI 0.98 to 1.41)	1.19 (95% CI 0.99 to 1.43)	
	Zika second trimester	31,339.29	1,814	282	9.00 (95% CI 7.99 to 10.09)	1.02 (95% CI 0.91 to 1.14)	1.03 (95% CI 0.91 to 1.16)	
	Zika third trimester	6,659.00	1,151	176	26.43 (95% CI 22.72 to 30.53)	1.06 (95% CI 0.91 to 1.23)	1.09 (95% CI 0.94 to 1.26)	
Congenital anomalies	Unexposed	268,509,857.86	6,973,030	66,004	0.25 (95% CI 0.24 to 0.25)			0.86
	Chikungunya first trimester	21,203.86	717	8	0.38 (95% CI 0.17 to 0.70)	1.09 (95% CI 0.54 to 2.17)	1.25 (95% CI 0.63 to 2.50)	
	Chikungunya second trimester	24,173.86	1,395	7	0.29 (95% CI 0.12 to 0.56)	0.50 (95% CI 0.24 to 1.05)	0.60 (95% CI 0.29 to 1.26)	
	Chikungunya third trimester	6,042.14	1,032	16	2.65 (95% CI 1.55 to 4.17)	1.67 (95% CI 1.02 to 2.73)	1.91 (95% CI 1.17 to 3.11)	
	Dengue first trimester	57,454.57	1,888	17	0.30 (95% CI 0.18 to 0.46)	0.97 (95% CI 0.60 to 1.57)	1.02 (95% CI 0.63 to 1.64)	
	Dengue second trimester	45,477.71	2,610	22	0.48 (95% CI 0.31 to 0.71)	0.92 (95% CI 0.61 to 1.40)	1.00 (95% CI 0.66 to 1.51)	
	Dengue third trimester	10,602.29	1,818	19	1.79 (95% CI 1.10 to 2.72)	1.23 (95% CI 0.78 to 1.93)	1.37 (95% CI 0.87 to 2.15)	
	Zika first trimester	19,935.43	694	42	2.11 (95% CI 1.53 to 2.81)	6.85 (95% CI 5.07 to 9.27)	9.01 (95% CI 6.65 to 12.21)	
	Zika second trimester	31,339.29	1,814	11	0.35 (95% CI 0.18 to 0.60)	0.65 (95% CI 0.36 to 1.18)	0.79 (95% CI 0.44 to 1.43)	
	Zika third trimester	6,659.00	1,151	10	1.50 (95% CI 0.75 to 2.63)	1.01 (95% CI 0.54 to 1.88)	1.19 (95% CI 0.64 to 2.21)	
Low Apgar 5'	Unexposed	265,467,842.86	6,893,512	61,905	0.23 (95% CI 0.23 to 0.24)			0.77
	Chikungunya first trimester	21,038.00	711	4	0.19 (95% CI 0.06 to 0.44)	0.58 (95% CI 0.22 to 1.55)	0.69 (95% CI 0.26 to 1.84)	
	Chikungunya second trimester	24,089.57	1,390	18	0.75 (95% CI 0.45 to 1.15)	1.41 (95% CI 0.89 to 2.24)	1.50 (95% CI 0.94 to 2.39)	
	Chikungunya third trimester	5,996.14	1,022	7	1.17 (95% CI 0.50 to 2.26)	0.95 (95% CI 0.45 to 1.99)	1.01 (95% CI 0.48 to 2.12)	
	Dengue first trimester	56,931.86	1,870	17	0.30 (95% CI 0.18 to 0.46)	1.03 (95% CI 0.64 to 1.66)	1.19 (95% CI 0.74 to 1.91)	
	Dengue second trimester	45,123.00	2,591	24	0.53 (95% CI 0.35 to 0.77)	1.10 (95% CI 0.73 to 1.64)	1.23 (95% CI 0.82 to 1.83)	
	Dengue third trimester	10,466.57	1,792	23	2.20 (95% CI 1.42 to 3.22)	1.99 (95% CI 1.32 to 2.99)	2.24 (95% CI 1.49 to 3.36)	
	Zika first trimester	19,857.14	691	11	0.55 (95% CI 0.29 to 0.95)	1.88 (95% CI 1.04 to 3.39)	2.03 (95% CI 1.12 to 3.70)	
	Zika second trimester	31,208.00	1,805	13	0.42 (95% CI 0.23 to 0.69)	0.84 (95% CI 0.49 to 1.45)	0.93 (95% CI 0.54 to 1.60)	
	Zika third trimester	6,631.86	1,148	9	1.36 (95% CI 0.65 to 2.45)	1.20 (95% CI 0.62 to 2.31)	1.29 (95% CI 0.67 to 2.47)	

*The estimated hazard for a model with time-varying covariates does not exhibit the same direct relationship with cumulative incidence as the standard Cox model. This discrepancy arises because cumulative incidence depends on the complete history of the time-varying covariate for all patients. Consequently, the hazard ratio estimates from these models will not correspond to a crude rate ratio calculated using events per person-time. CI = Robust Confidence Interval

Supplementary Table 7: Estimated risk ratio of neonatal death (<28 days) by exposure in the women with confirmed symptomatic infection by clinical or laboratory diagnosis. Analysis using missing indicator.

	No. pregnancies	*Events (%)*	*Adjusted Risk Ratio*	*Risk per 10,000 pregnancies*	*Marginal Risk Difference per 10,000 pregnancies*
Unexposed	6,959,911	43,756 (0.63%)		62.44 (95% CI 52.16 to 72.72)	
Chikungunya overall	6,066	53(0.87%)	1.50 (95% CI 1.16 to 1.94)	93.84 (95% CI 65.11 to 122.57)	31.40 (95% CI 6.09 to 55.92)
Chikungunya first trimester	1,534	9 (0.59%)	1.03 (95% CI 0.52 to 2.03)	64.31 (95% 53.13 to 106.49)	1.88 (95% CI -41.66 to 45.42)
Chikungunya second trimester	2,529	26 (1.03%)	1.74 (95% CI 1.28 to 2.38)	108.69 (95% CI 68.41 to 148.97)	46.41 (95% CI 10.74 to 82.08)
Chikungunya third trimester	2,003	18 (0.90%)	1.55 (95% CI 1.03 to 2.32)	96.72 (95% CI 53.13 to 140.31)	34.33 (95% CI -5.67 to 74.32)
Dengue overall	19,022	118(0.62%)	1.10 (95% CI 0.93 to 1.29)	68.67 (95% CI 53.36 to 83.97)	6.23 (95% CI -4.93 to 17.38)
Dengue first trimester	6,847	36 (0.53%)	0.93 (95% CI 0.68 to 1.27)	58.06 (95% CI 39.12 to 77.00)	-4.39 (95% CI -22.45 to 13.67)
Dengue second trimester	7,174	51 (0.71%)	1.29 (95% CI 0.99 to 1.67)	80.43 (95% CI 55.48 to 105.38)	18.03 (95% CI -3.13 to 39.19)
Dengue third trimester	5,001	31 (0.62%)	1.07 (95% CI 0.73 to 1.56)	66.72 (95% CI 39.22 to 94.21)	4.28 (95% CI -20.96 to 29.53)
Zika overall	8,396	51 (0.61%)	1.09 (95% CI 0.85 to 1.40)	68.39 (95% CI 48.69 to 88.09)	5.95 (95% CI -10.95 to 22.86)
Zika first trimester	1,810	18 (0.99%)	1.72 (95% CI 1.12to 2.62)	107.09 (95% CI 59.62 to 154.56)	44.69 (95% CI -0.675 to 90.05)
Zika second trimester	3,750	22 (0.59%)	1.07 (95% CI 0.72 to 1.59)	66.86 (95% CI 38.57 to 95.14)	4.41 (95% CI -21.99 to 30.80)
Zika third trimester	2,836	12 (0.42%)	0.74 (95% CI 0.48 to 1.15)	46.53 (95% CI 25.40 to 67.65)	-15.93 (95% CI -36.34 to 4.48)

*The 95% Confidence Interval (CI) of the Risk and Risk Difference was estimated using the delta method.

Supplementary Table 8: Estimated risk ratio of neonatal death (<28 days) by exposure in the women with confirmed symptomatic infection only by laboratory diagnosis. Analysis using missing indicator.

	No. pregnancies	*Events (%)*	*Adjusted Risk Ratio*	*Risk per 10,000 pregnancies*	*Marginal Risk Difference per 10,000 pregnancies*
Unexposed	6,959,911	43,756 (0.63%)		62.45 (95% CI 52.21 to 72.69)	
Chikungunya overall	3,155	25(0.79%)	1.44 (95% CI 1.00 to 2.07)	89.64 (95% CI 54.19 to 125.09)	27.19 (95% CI -5.60 to 59.98)

Chikungunya first trimester	719	3(0.42%)	0.82 (95% CI 0.28 to 2.42)	51.44 (95% CI 0 to 106.40)	-11.01 (95% CI -66.70 to 44.69)
Chikungunya second trimester	1,400	12(0.86%)	1.53 (95% CI 0.87 to 2.67)	95.39 (95% CI 41.91 to 148.87)	32.94 (95% CI -19.91 to 85.76)
Chikungunya third trimester	1,036	10(0.97%)	1.69 (95% CI 0.87 to 3.27)	105.50 (95% CI 30.75 to 180.25)	43.05 (95% CI -28.16 to 114.27)
Dengue overall	6,316	45(0.71%)	1.42 (95% CI 1.05 to 1.90)	88.47 (95% CI 59.24 to 117.70)	26.02 (95% CI 0.21 to 52.24)
Dengue first trimester	1,888	12(0.64%)	1.27 (95% CI 0.72 to 2.23)	79.25 (95% CI 33.89 to 124.60)	16.80 (95% CI -27.69 to 61.28)
Dengue second trimester	2,610	18(0.69%)	1.39 (95% CI 0.89 to 2.17)	86.94 (95% CI 46.06 to 127.82)	24.49 (95% CI -14.38 to 63.37)
Dengue third trimester	1,818	15(0.83%)	1.60 (95% CI 0.95 to 2.69)	99.85 (95% CI 44.81 to 154.89)	37.40 (95% CI -15.16 to 89.96)
Zika overall	3,674	17(0.46%)	0.88 (95% CI 0.53 to 1.45)	54.98 (95% CI 26.61 to 83.35)	-7.47 (95% CI -35.09 to 20.16)
Zika first trimester	697	6(0.86%)	1.69 (95% CI 0.65 to 4.39)	105.51 (95% CI 3.78 to 207.24)	43.06 (95% CI -57.65 to 143.77)
Zika second trimester	1,819	9(0.49%)	0.95 (95% CI 0.51 to 1.72)	59.14 (95% CI 23.14 to 95.14)	-3.31 (95% CI -38.76 to 32.15)
Zika third trimester	1,158	2(0.17%)	0.32 (95% CI 0.07 to 1.39)	19.96 (95% CI 0 to 49.37)	-42.49 (95% CI -72.66 to -12.30)

*The 95% Confidence Interval (CI) of the Risk and Risk Difference was estimated using the delta method.

Supplementary Table 9: Estimated adjusted hazard ratios (birth outcomes) and adjusted risk ratios (neonatal death) using complete case analysis.

Outcomes	N	EVENTS	ESTIMATE (95% CONFIDENCE INTERVAL)
**Preterm**			
Unexposed	6297566	617437	
Chikungunya	4713	467	1.11 (1.01 to 1.21)
Dengue	16427	1519	1.06 (1.01 to 1.12)
Zika	7121	722	1.24 (1.15 to 1.33)
**Low birth weight**			
Unexposed	6297566	466518	
Chikungunya	4983	337	1.14 (1.03 to 1.27)
Dengue	17057	1236	1.08 (1.03 to 1.15)
Zika	7534	577	1.27 (1.17 to 1.38)

**Small for gestational age**			
Unexposed	6297566	433117	
Chikungunya	4983	388	1.09 (0.99 to 1.21)
Dengue	17057	1272	0.99 (0.94 to 1.05)
Zika	7534	592	1.2 (1.11 to 1.3)
**Large for gestational age**			
Unexposed	6297566	973095	
Chikungunya	4983	861	1.01 (0.95 to 1.08)
Dengue	17057	2293	0.93 (0.9 to 0.97)
Zika	7534	1259	1.12 (1.06 to 1.19)
**Congenital anomalies**			
Unexposed	6297566	60185	
Chikungunya	4983	45	1.11 (0.83 to 1.49)
Dengue	17057	167	1.14 (0.98 to 1.33)
Zika	7534	119	2.18 (1.82 to 2.62)
**Low Apgar 5'**			
Unexposed	6297566	55011	
Chikungunya	4983	60	1.54 (1.19 to 1.98)
Dengue	17057	159	1.22 (1.04 to 1.43)
Zika	7534	68	1.2 (0.94 to 1.52)
**Neonatal death**			
Unexposed	6297566	38180	
Chikungunya	4983	45	1.59 (1.19 to 2.13)
Dengue	17057	97	1.04 (0.85 to 1.27)
Zika	7534	42	1.03 (0.76 to 1.4)

Confidence Interval = Robust Confidence Interval

Supplementary Table 10: Distribution of congenital anomalies by exposure.

Block from ICD-10 Chapter XVII	Unexposed, N = 6 ,959, 911	Chikungunya, N = 6, 066	Dengue, N = 19 ,022	Zika, N = 8 ,396
Congenital malformations of the nervous system	8 ,908 (0.1%)	15 (0.2%)	57 (0.3%)	84 (1.0%)
Congenital malformations of eye, ear, face and neck	5 ,919 (0.1%)	5 (0.1%)	16 (0.1%)	10 (0.1%)
Congenital malformations of the circulatory system	8 ,487 (0.1%)	6 (0.1%)	11 (0.1%)	9 (0.1%)
Congenital malformations of the respiratory system	8 ,404 (0.1%)	5 (0.1%)	21 (0.1%)	10 (0.1%)
Cleft lip and cleft palate	8 ,404 (0.1%)	5 (0.1%)	21 (0.1%)	10 (0.1%)
Other congenital malformations of the digestive system	9 ,842 (0.1%)	6 (0.1%)	24 (0.1%)	10 (0.1%)
Congenital malformations of genital organs	5 ,469 (0.1%)	<5 (<0.1%)	16 (0.1%)	<5 (<0.1%)
Congenital malformations of the urinary system	21 ,012 (0.3%)	15 (0.2%)	61 (0.3%)	22 (0.3%)
Congenital malformations and deformations of the musculoskeletal system	29 ,369 (0.4%)	23 (0.4%)	85 (0.4%)	36 (0.4%)
Other congenital malformations	3, 893 (0.1%)	<5 (<0.1%)	7 (0.0%)	8 (0.1%)
Chromosomal abnormalities, not elsewhere classified	3 ,832 (0.1%)	5 (0.1%)	10 (0.1%)	<5 (<0.1%)

Chikungunya Infection

Block from ICD-10 Chapter XVII	Chikungunya-first, N = 1,534	Second, N = 2,529	Third, N = 2,003
Congenital malformations of the nervous system	<5 (<0.3%)	6(0.2%)	6 (0.3%)
Congenital malformations of eye, ear, face and neck	0 (0%)	<5 (<0.2%)	<5 (<0.3%)
Congenital malformations of the circulatory system	<5 (<0.3%)	<5 (<0.2%)	<5 (<0.3%)
Congenital malformations of the respiratory system	<5 (<0.3%)	<5 (<0.2%)	<5 (<0.3%)
Cleft lip and cleft palate	<5 (<0.3%)	<5 (<0.2%)	<5 (<0.3%)
Other congenital malformations of the digestive system	<5 (<0.3%)	<5 (<0.2%)	<5 (<0.3%)
Congenital malformations of genital organs	<5 (<0.3%)	0 (0%)	<5 (<0.3%)
Congenital malformations of the urinary system	<5 (<0.3%)	6 (0.2%)	6 (0.3%)
Congenital malformations and deformations of the musculoskeletal system	<5 (<0.3%)	9 (0.4%)	10 (0.5%)
Other congenital malformations	<5 (<0.3%)	0 (0.0%)	<5 (<0.3%)

Chromosomal abnormalities, not elsewhere classified	<5 (<0.3%)	0 (0.0%)	<5 (<0.3%)

Dengue Infection

Block from ICD-10 Chapter XVII	Dengue-first, N = 6,847	Second, N = 7,175	Third, N = 5,000
Congenital malformations of the nervous system	31 (0.5%)	11(0.2%)	15 (0.3%)
Congenital malformations of eye, ear, face and neck	5 (0.1%)	9 (0.1%)	<5 (<0.1%)
Congenital malformations of the circulatory system	<5 (<0.1%)	5 (0.1%)	<5 (<0.1%)
Congenital malformations of the respiratory system	8 (0.1%)	11 (0.2%)	<5 (<0.1%)
Cleft lip and cleft palate	8 (0.1%)	11 (0.2%)	<5 (<0.1%)
Other congenital malformations of the digestive system	8 (0.1%)	12 (0.2%)	<5 (<0.1%)
Congenital malformations of genital organs	<5 (<0.1%)	9 (0.1%)	<5 (<0.1%)
Congenital malformations of the urinary system	24 (0.4%)	19 (0.3%)	18 (0.4%)
Congenital malformations and deformations of the musculoskeletal system	37 (0.5%)	23 (0.4%)	25 (0.4%)
Other congenital malformations	<5 (<0.1%)	<5 (<0.1%)	<5 (<0.1%)
Chromosomal abnormalities, not elsewhere classified	5 (0.1%)	<5 (<0.1%)	<5 (<0.1%)

Zika Infection

Block from ICD-10 Chapter XVII	Zika-first, N = 1,810	Second, N = 3,750	Third, N = 2,836
Congenital malformations of the nervous system	62 (3.4%)	8 (0.2%)	14 (0.5%)
Congenital malformations of eye, ear, face and neck	5 (0.3%)	<5 (<0.2%)	<5 (<0.2%)
Congenital malformations of the circulatory system	5 (0.3%)	<5 (<0.2%)	0 (0.0%)
Congenital malformations of the respiratory system	<5 (<0.2%)	5 (0.1%)	<5 (<0.2%)
Cleft lip and cleft palate	<5 (<0.2%)	5 (0.1%)	<5 (<0.2%)
Other congenital malformations of the digestive system	<5 (<0.2%)	5 (0.1%)	<5 (<0.2%)
Congenital malformations of genital organs	<5 (<0.2%)	<5 (<0.2%)	<5 (<0.2%)
Congenital malformations of the urinary system	6 (0.3%)	8 (0.2%)	8 (0.3%)
Congenital malformations and deformations of the musculoskeletal system	14 (0.8%)	13 (0.3%)	9 (0.3%)
Other congenital malformations	<5 (<0.2%)	<5 (<0.2%)	<5 (<0.2%)

Chromosomal abnormalities, not elsewhere classified	0 (0%)	<5 (<0.2%)	0 (0%)

Supplementary Table 11: Distribution of deaths (ICD-10) by exposure.

**Block from ICD-10 Chapter XVII**	**Unexposed, N = 43,469**	**Chikungunya, N = 53**	**Dengue, N = 118**	**Zika, N = 51**
Certain conditions originating in the perinatal period	31422 (72%)	35 (66%)	84 (71%)	36 (71%)
Congenital malformations, deformations and chromosomal abnormalities	10694 (25%)	14 (26%)	30 (25%)	11 (21%)
Certain infectious and parasitic diseases	549 (1%)	<5 (<5%)	0	<5 (<5%)
External causes of morbidity and mortality	247 (0.5%)	<5 (<5%)	<5 (<4%)	<5 (<5%)
Symptoms, signs and abnormal clinical and laboratory findings, not elsewhere classified	172 (<0.5%)	<5 (<5%)	<5 (<4%)	0
Diseases of the respiratory system	159 (<0.5%)	0	<5 (<4%)	<5 (<5%)
**Others causes**	226 (0.5%)	0	0	0

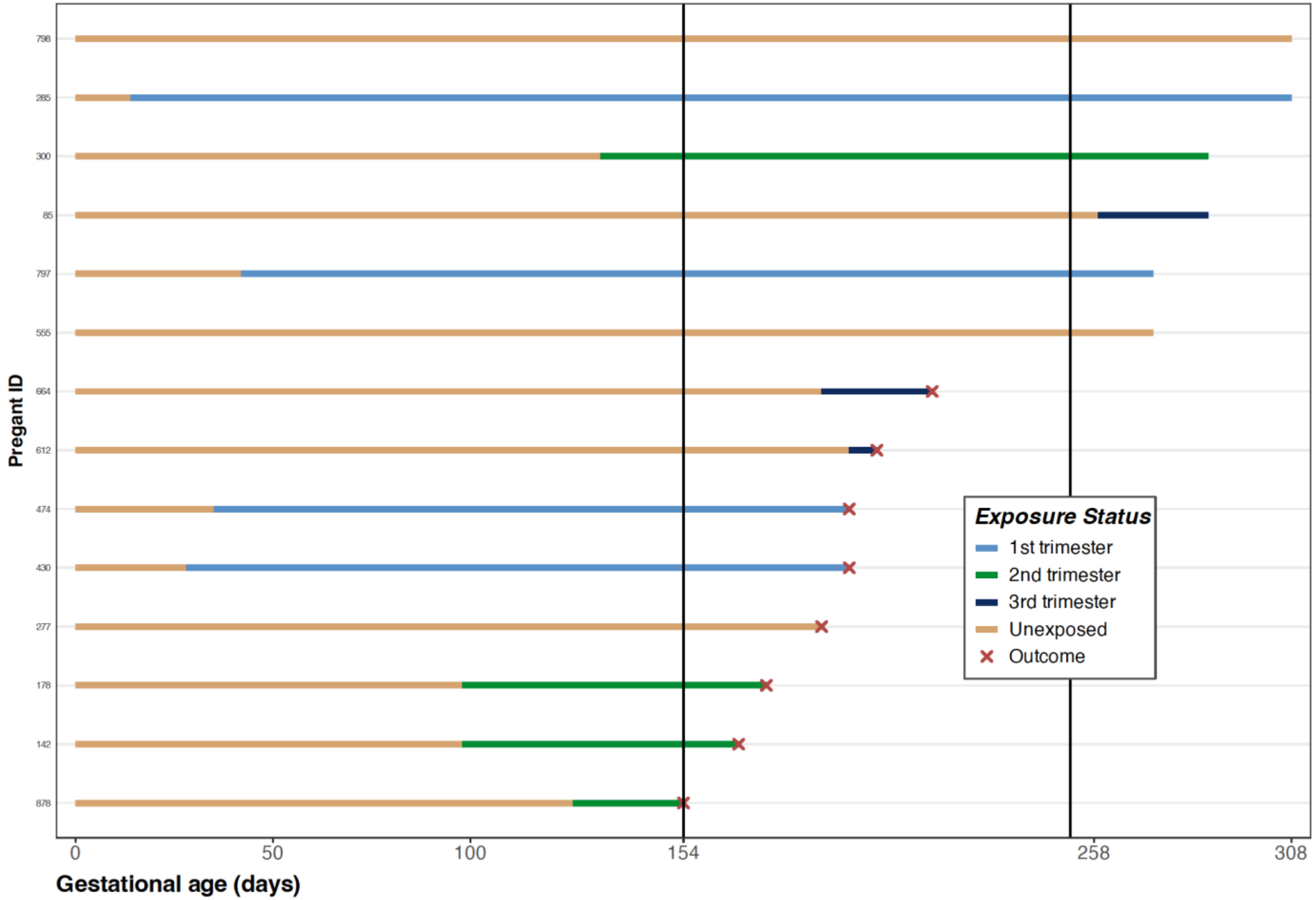

Supplementary Figure 1: Exposure timelines for preterm analysis. Each line represents one pregnancy, and the line's colour represents the infection status for one arbovirus (time-dependent exposure). A red X indicates occurrence of an adverse outcome

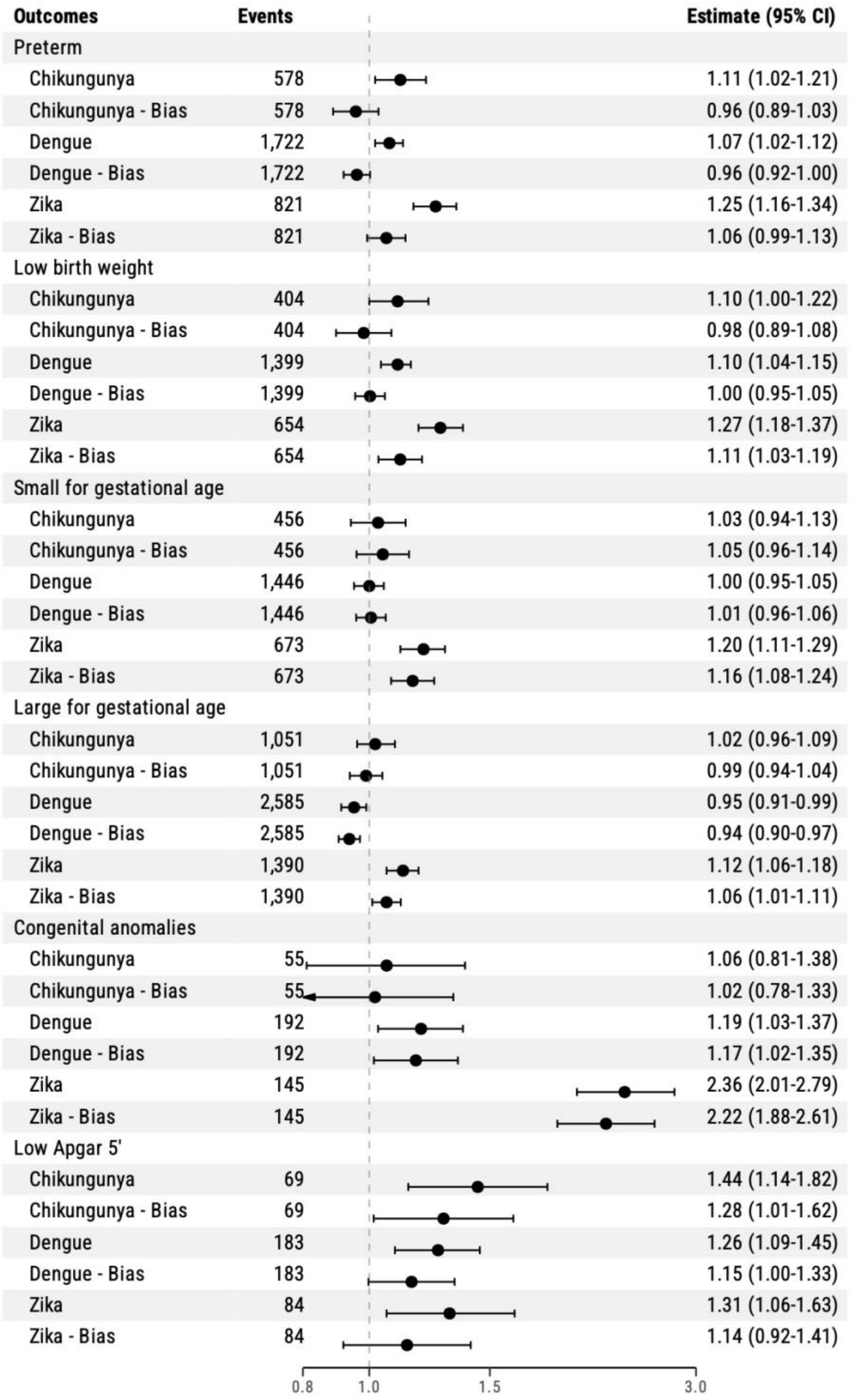

Supplementary Figure 2: Estimated adjusted hazard ratio (birth outcomes) and adjusted risk ratio (neonatal death), comparing groups exposed and unexposed to arbovirus infection during pregnancy by outcome. "Bias" estimates are derived from the model, classifying the live born as exposed since conception if their mother had an infection at any point during the pregnancy, i.e. not using time-varying exposure. Errors bars represent the 95% confidence interval. The numbers of individuals in each group are present in the Supplementary Table 3.

Supplementary Figure 3 – Performance Linkage. Linkage between the Live Birth information System and arbovirus notification information system

Chikungunya:<br>Best cutoff: 0.930<br>Sensitivity: 98.0<br>Specificity: 98.90	Dengue:<br>Best cutoff: 0.930<br>Sensitivity: 98.8<br>Specificity: 98.6	Zika<br>Best cutoff: 0.942<br>Sensitivity: 97.7<br>Specificity: 99.0
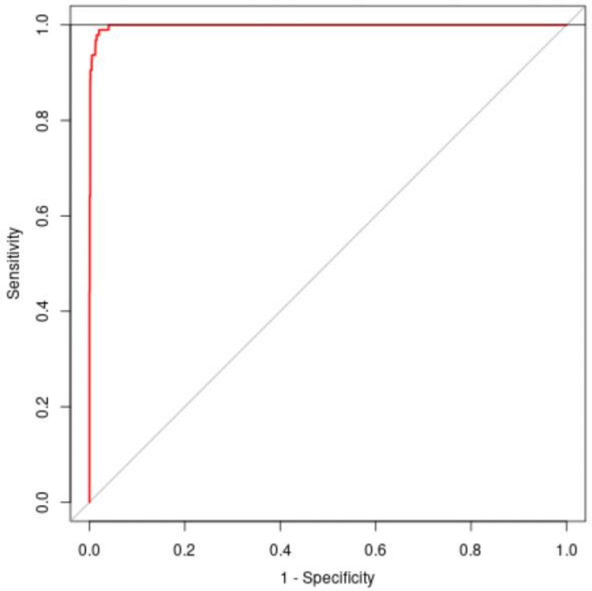	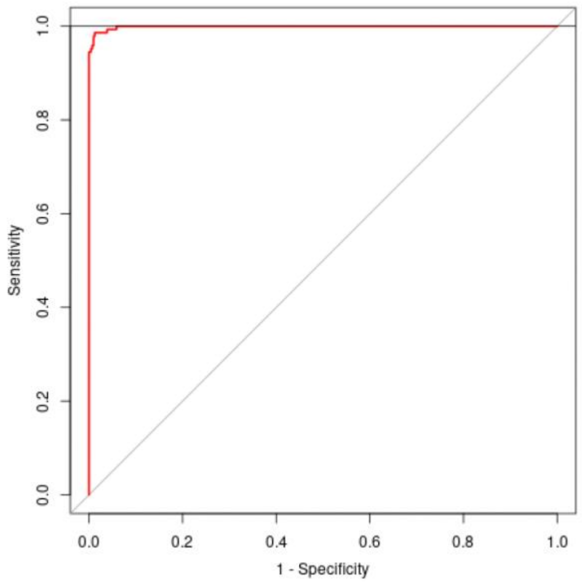	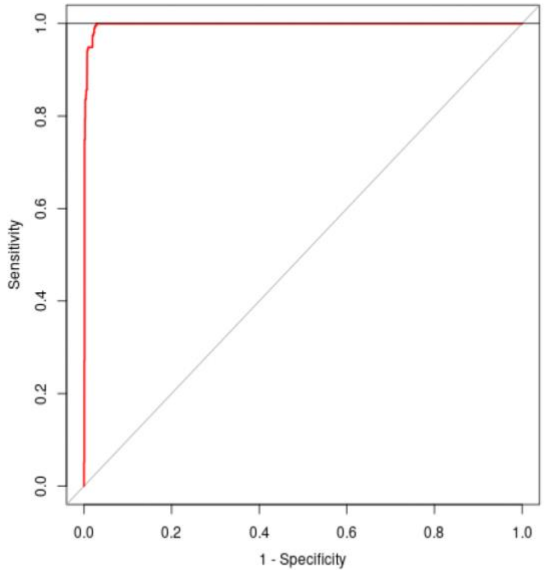

STROBE Statement—checklist of items that should be included in reports of observational studies

	Item No.	Recommendation	Location
**Title and abstract**	1	(*a*) Indicate the study's design with a commonly used term in the title or the abstract	Title
		(*b*) Provide in the abstract an informative and balanced summary of what was done and what was found	Abstract
**Introduction**			
Background/rationale	2	Explain the scientific background and rationale for the investigation being reported	4th/5th paragraph of introduction
Objectives	3	State specific objectives, including any prespecified hypotheses	6th paragraph of introduction
**Methods**			
Study design	4	Present key elements of study design early in the paper	
Setting	5	Describe the setting, locations, and relevant dates, including periods of recruitment, exposure, follow-up, and data collection	Methods section – "Study population and data sources"
Participants	6	(*a*) *Cohort study*—Give the eligibility criteria, and the sources and methods of selection of participants. Describe methods of follow-up<br>*Case-control study*—Give the eligibility criteria, and the sources and methods of case ascertainment and control selection. Give the rationale for the choice of cases and controls<br>*Cross-sectional study*—Give the eligibility criteria, and the sources and methods of selection of participants	Last paragraph of Methods section – "Study population and data sources"
		(*b*) *Cohort study*—For matched studies, give matching criteria and number of exposed and unexposed<br>*Case-control study*—For matched studies, give matching criteria and the number of controls per case	
Variables	7	Clearly define all outcomes, exposures, predictors, potential confounders, and effect modifiers. Give diagnostic criteria, if applicable	Methods section "Exposures and outcomes" and "Covariables" sections
Data sources/ measurement	8*	For each variable of interest, give sources of data and details of methods of assessment (measurement). Describe comparability of assessment methods if there is more than one group	*N/A*
Bias	9	Describe any efforts to address potential sources of bias	Methods section – "Sensitivity Analyses"
Study size	10	Explain how the study size was arrived at	Methods section – "Study population and data sources"
Quantitative variables	11	Explain how quantitative variables were handled in the analyses. If applicable, describe which groupings were chosen and why	N/A
Statistical methods	12	(*a*) Describe all statistical methods, including those used to control for confounding	Methods section – "Statistical Analyses"
		(*b*) Describe any methods used to examine subgroups and interactions	Methods section – "Statistical Analyses"
		(*c*) Explain how missing data were addressed	Methods section – "Covariables"
		(*d*) *Cohort study*—If applicable, explain how loss to follow-up was addressed<br>*Case-control study*—If applicable, explain how matching of cases and controls was addressed	Methods section – "Statistical Analyses"

		Cross-sectional study—If applicable, describe analytical methods taking account of sampling strategy	
		(*e*) Describe any sensitivity analyses	Methods section – "Sensitivity Analyses"
**Results**			
Participants	13*	(a) Report numbers of individuals at each stage of study—eg numbers potentially eligible, examined for eligibility, confirmed eligible, included in the study, completing follow-up, and analysed	Results section – "first paragraph"
		(b) Give reasons for non-participation at each stage	Results section – "Figure 1"
		(c) Consider use of a flow diagram	Results section – "Figure 1"
Descriptive data	14*	(a) Give characteristics of study participants (eg demographic, clinical, social) and information on exposures and potential confounders	Results section – Table 1 and Supplementary Table 1
		(b) Indicate number of participants with missing data for each variable of interest	Results section – Table 1 and Supplementary Table 1
		(c) *Cohort study*—Summarise follow-up time (eg, average and total amount)	Supplementary Table 2 to 6
Outcome data	15*	*Cohort study*—Report numbers of outcome events or summary measures over time	Results section – Table 1 and Supplementary Table 1
		Case-control study—Report numbers in each exposure category, or summary measures of exposure	
		Cross-sectional study—Report numbers of outcome events or summary measures	
Main results	16	(*a*) Give unadjusted estimates and, if applicable, confounder-adjusted estimates and their precision (eg, 95% confidence interval). Make clear which confounders were adjusted for and why they were included	Supplementary Table 2 to 6
		(*b*) Report category boundaries when continuous variables were categorized	Methods section - covariables
		(*c*) If relevant, consider translating estimates of relative risk into absolute risk for a meaningful time period	Supplementary Tables 7 and 8

Continued on next page

Other analyses	17	Report other analyses done—eg analyses of subgroups and interactions, and sensitivity analyses	Supplementary Tables 4/6/8
**Discussion**			
Key results	18	Summarise key results with reference to study objectives	Discussion section – first paragraph
Limitations	19	Discuss limitations of the study, taking into account sources of potential bias or imprecision. Discuss both direction and magnitude of any potential bias	Discussion section – sixth paragraph
Interpretation	20	Give a cautious overall interpretation of results considering objectives, limitations, multiplicity of analyses, results from similar studies, and other relevant evidence	Discussion section – sixth paragraph
Generalisability	21	Discuss the generalisability (external validity) of the study results	Discussion section – seventh paragraph
**Other information**			
Funding	22	Give the source of funding and the role of the funders for the present study and, if applicable, for the original study on which the present article is based	Funding section

*Give information separately for cases and controls in case-control studies and, if applicable, for exposed and unexposed groups in cohort and cross-sectional studies.
