# Supplementary material for: Perinatal outcomes of symptomatic chikungunya, dengue and Zika infection during pregnancy in Brazil: a registry-based cohort study
Source: Nat Commun. 2025 Aug 5;16:7207. doi: 10.1038/s41467-025-62640-x (PMC12325709; doi:10.1038/s41467-025-62640-x)
Supplement: Supplementary file 3 — Reporting Summary [file 41467_2025_62640_MOESM3_ESM.pdf]

nature portfolio

Corresponding author(s): Thiago Cerqueira Silva

Last updated by author(s): Jul 10, 2025

Reporting Summary

Nature Portfolio wishes to improve the reproducibility of the work that we publish. This form provides structure for consistency and transparency in reporting. For further information on Nature Portfolio policies, see our Editorial Policies and the Editorial Policy Checklist.

Statistics

For all statistical analyses, confirm that the following items are present in the figure legend, table legend, main text, or Methods section.

n/a	Confirmed	
[ ]	[x]	The exact sample size (*n*) for each experimental group/condition, given as a discrete number and unit of measurement
[ ]	[x]	A statement on whether measurements were taken from distinct samples or whether the same sample was measured repeatedly
[x]	[ ]	The statistical test(s) used AND whether they are one- or two-sided<br>*Only common tests should be described solely by name; describe more complex techniques in the Methods section.*
[ ]	[x]	A description of all covariates tested
[ ]	[x]	A description of any assumptions or corrections, such as tests of normality and adjustment for multiple comparisons
[ ]	[x]	A full description of the statistical parameters including central tendency (e.g. means) or other basic estimates (e.g. regression coefficient) AND variation (e.g. standard deviation) or associated estimates of uncertainty (e.g. confidence intervals)
[ ]	[x]	For null hypothesis testing, the test statistic (e.g. *F*, *t*, *r*) with confidence intervals, effect sizes, degrees of freedom and *P* value noted<br>*Give P values as exact values whenever suitable.*
[x]	[ ]	For Bayesian analysis, information on the choice of priors and Markov chain Monte Carlo settings
[x]	[ ]	For hierarchical and complex designs, identification of the appropriate level for tests and full reporting of outcomes
[x]	[ ]	Estimates of effect sizes (e.g. Cohen's *d*, Pearson's *r*), indicating how they were calculated

Our web collection on statistics for biologists contains articles on many of the points above.

Software and code

Policy information about availability of computer code

Data collection	R version 4.1.1
Data analysis	The modelling in this paper used R v.4.1.1 and the tidyverse v.1.3.2, survival v.3.7-0, mice v.3.17, fixest v.0.11.2, marginaleffects 0.18.0 R packages, all of which are freely available

For manuscripts utilizing custom algorithms or software that are central to the research but not yet described in published literature, software must be made available to editors and reviewers. We strongly encourage code deposition in a community repository (e.g. GitHub). See the Nature Portfolio guidelines for submitting code & software for further information.

Data

Policy information about availability of data

All manuscripts must include a data availability statement. This statement should provide the following information, where applicable:

- Accession codes, unique identifiers, or web links for publicly available datasets
- A description of any restrictions on data availability
- For clinical datasets or third party data, please ensure that the statement adheres to our policy

The relevant data are available in the manuscript and the Supplementary Information. Raw data are available upon reasonable request to the Centro de Integração de Dados e Conhecimentos para a Saúde (CIDACS). Any person who wishes to receive authorisation must: (1) be affiliated to CIDACS or be accepted as collaborators; (2) present a detailed research project together with approval by an appropriate Brazilian institutional research ethics committee; (3) provide a clear data plan restricted to the objectives of the proposed study and a summary of the analyses plan intended to guide the linkage and data extraction of the relevant

set of records and variables; (4) sign terms of responsibility regarding the access and use of data; and (5) perform the analyses of datasets provided using the CIDACS data environment, a safe and secure infrastructure that provides remote access to de-identified or anonymised datasets and analysis tools. For more information: https://cidacs.bahia.fiocruz.br/

Research involving human participants, their data, or biological material

Policy information about studies with human participants or human data. See also policy information about sex, gender (identity/presentation), and sexual orientation and race, ethnicity and racism.

Reporting on sex and gender	We used sex (biological attribute) when describing the sex of the live births. Otherwise, all the sample is composed of pregnant women.
Reporting on race, ethnicity, or other socially relevant groupings	We used race (classified in accordance to the IBGE - Instituto Brasileiro de Geografia e Estatística), white/black/mixed/asian-yellow/indigenous. In previous research, it was associated different rates of adverse outcomes for women of different races.
Population characteristics	All the information avaliable in the database is presented in the Table 1 of the manuscrirpt
Recruitment	This is a observational study using data from national administrative databases
Ethics oversight	The Federal University of Bahia's Institute of Public Health Ethics Committee approved the study (CAAE registration number: 18022319.4.0000.5030).

Note that full information on the approval of the study protocol must also be provided in the manuscript.

Field-specific reporting

Please select the one below that is the best fit for your research. If you are not sure, read the appropriate sections before making your selection.

[x] Life sciences [ ] Behavioural & social sciences [ ] Ecological, evolutionary & environmental sciences

For a reference copy of the document with all sections, see nature.com/documents/nr-reporting-summary-flat.pdf

Life sciences study design

All studies must disclose on these points even when the disclosure is negative.

Sample size	Given that the sample sizes were based on national linked datasets and our aim is to estimate the effects of arbovirus in perinatal outcomes. We considered that the resulting 6,993,395 live births was sufficient.
Data exclusions	We excluded: (i) births that took place prior to 22 weeks or after 44 weeks of gestation, or with a birthweight below 500g; (ii) non-singleton live births; (iii) live births with missing data on municipality of residence; (iv) live births from women aged less than 12 years or older than 49. Additionally, for the exposed women, we excluded live births from women with more than one confirmed arbovirus infection during pregnancy; and for the unexposed live births, we excluded women with suspected arbovirus infection during pregnancy without clinical or laboratory confirmation, as those records are likely to be cases of other febrile diseases
Replication	We repeated the analysis using pregnancies with confirmed arbovirus infection through laboratory diagnosis. We also repeated the analysis using missing indicator instead of multiple imputation for missing variables
Randomization	Not applicable (observational study)
Blinding	Not applicable (observational study)

Reporting for specific materials, systems and methods

We require information from authors about some types of materials, experimental systems and methods used in many studies. Here, indicate whether each material, system or method listed is relevant to your study. If you are not sure if a list item applies to your research, read the appropriate section before selecting a response.

Materials & experimental systems

n/a	Involved in the study
☒	☐ Antibodies
☒	☐ Eukaryotic cell lines
☒	☐ Palaeontology and archaeology
☒	☐ Animals and other organisms
☒	☐ Clinical data
☒	☐ Dual use research of concern
☒	☐ Plants

Methods

n/a	Involved in the study
☒	☐ ChIP-seq
☒	☐ Flow cytometry
☒	☐ MRI-based neuroimaging

Plants

Seed stocks	N/A
Novel plant genotypes	N/A
Authentication	N/A
